# Personalities

**Published:** 1892-11-05

**Authors:** 


### PASSING TOPICS.

### PERSONALITIES.

Has not the time come when a protest may be entered with advantage against the prevalence of the personal element in the discussions which arise regarding matters profoundly impersonal? It has often been said, and truly, that abuse is not argument, while it is presumptive evidence of a bad case. We do not suggest that people of divergent sentiments and convictions should meekly "agree to differ." On the contrary, however well suited that arrangement may be to the amenities of private life, it is well that public and institutional questions should be debated fully and without reserve, but also without acrimony, and certainly without calling names.

The better the cause the worse the effect of mere wrangling. We need not travel beyond the last few weeks to learn the effect of mere blustering and raving upon the public mind, and how a strong case may be weakened by unworthy advocacy. Nothing has created so many difficulties in the way of accepting religious truth, whether in the savage or the civilised mind, as the rancorous animosities developed by rival sects—all professing the gospel of peace. Similarly we can imagine few things more likely to arouse disappointment and even a stronger feeling in onlookers than the license taken by a certain class of hospital critics and hospital workers to attribute unholy motives to everyone who dissents from them on controversial subjects. There are many questions in the hospital world about which a difference of opinion is not only reasonable, but may be beneficial, if only the argument be temperately conducted.

Of course, a homily is not likely to be brilliant, and we are fully alive to the fact that all we can say upon this subject is commonplace enough. It is the merest truism to assert that emphasis is not the less emphatic because it is expressed without virulence, and we cannot believe that any cause is likely to be served by language studiously disdainful or insulting. No one can deny that political life has been distinctly debased of recent years, in the opinion not only of the public, but of the best examples of both parties, by the common occurrence of disgraceful incidents, and that there is a sensible decline in the standard of political morality concurrent with, and doubtless attributable, to the deterioration in manners.

In the same way nothing is calculated to do more harm to hospital work than persistence in the spiteful method of discussion which has been so rife. One result is a perceptible loss of confidence in each other among different classes of workers. Æsop's old fable of the revolt of the limbs against the head is promised a fresh illustration, and, unless the good sense of the majority prevails, we may have to add to the other difficulties of hospital administration, a want of cohesion among the workers which, more that anything else, is calculated to weaken the fabric of the voluntary system, and to afford assistance to those who unhappily are only too ready to take a part in its demolition.

Agricola.

